# Impact of Dietary Supplementation of *Lactic Acid Bacteria* Fermented Rapeseed with or without Macroalgae on Performance and Health of Piglets Following Omission of Medicinal Zinc from Weaner Diets

**DOI:** 10.3390/ani10010137

**Published:** 2020-01-15

**Authors:** Gizaw D. Satessa, Paulina Tamez-Hidalgo, Yan Hui, Tomasz Cieplak, Lukasz Krych, Søren Kjærulff, Grete Brunsgaard, Dennis S. Nielsen, Mette O. Nielsen

**Affiliations:** 1 Department of Veterinary and Animal Sciences, University of Copenhagen, Grønnegårdsvej 3, 1870 Frederiksberg C, Denmark; gizaw.satessa@sund.ku.dk; 2 Fermentationexperts A/S, Vorbassevej 12, 6622 Copenhagen, Denmark; pat@fexp.eu (P.T.-H.); skj@fermbiotics.com (S.K.); grb@fexp.eu (G.B.); 3 Department of Food Science, University of Copenhagen, Rolighedsvej 26, 1958 Frederiksberg C, Denmark; huiyan@food.ku.dk (Y.H.); DKTOCI@chr-hansen.com (T.C.); krych@food.ku.dk (L.K.); dn@food.ku.dk (D.S.N.); 4 Department of Animal Sciences, Faculty of Technical Sciences, Aarhus University, Blichers Allé 20, 8830 Tjele, Denmark

**Keywords:** jejunal villus development, gut barrier function, colon microbiota, *Ascophyllum nodossum*, *Saccharina latissima*

## Abstract

**Simple Summary:**

Weaning is the most stressful event in pig production and is often associated with reduced performance, diarrhoea and piglet mortality. Currently, a high dose of zinc oxide (ZnO) is used to prevent weaning-related loss in productivity. However, the feeding of ZnO in weaner piglets will be phased out by 2022 in Europe, leaving pig producers without options to manage post-weaning disorders. This study investigated whether fermented rapeseed meal (FRM) alone or in combination with one (FRMA) or more (FRMAS) brown macroalgae species could improve weaner piglet growth, intestinal development and health compared to either non-supplemented diets (negative control, NC) or diets supplemented with 2500 ppm ZnO (positive control, PC). Both FRM and FRMA resulted in a similar production performance to PC when fed to weaned piglets. The PC, FRM and FRMAS (gender-specific) improved jejunal villus development more than the NC. Colon mucosal development was stimulated, and signs of intestinal inflammation were reduced by FRM. The composition and diversity of colon microbiota were similar between all fermented feeds and PC, but different compared to NC. In conclusion, FRM was at least as effective as ZnO to improve piglet growth, intestinal development and health.

**Abstract:**

The feeding of medicinal zinc oxide (ZnO) to weaner piglets will be phased out after 2022 in Europe, leaving pig producers without options to manage post-weaning disorders. This study assessed whether rapeseed meal, fermented alone (FRM) or co-fermented with a single (*Ascophylum nodosum*; FRMA), or two (*A. nodossum* and *Saccharina latissima*; FRMAS) brown macroalagae species, could improve weaner piglet performance and stimulate intestinal development as well as maturation of gut microbiota in the absence of in-feed zinc. Weaned piglets (n = 1240) were fed, during 28–85 days of age, a basal diet with no additives (negative control; NC), 2500 ppm in-feed ZnO (positive control; PC), FRM, FRMA or FRMAS. Piglets fed FRM and FRMA had a similar or numerically improved, respectively, production performance compared to PC piglets. Jejunal villus development was stimulated over NC in PC, FRM and FRMAS (gender-specific). FRM enhanced colon mucosal development and reduced signs of intestinal inflammation. All fermented feeds and PC induced similar changes in the composition and diversity of colon microbiota compared to NC. In conclusion, piglet performance, intestinal development and health indicators were sustained or numerically improved when in-feed zinc was replaced by FRM.

## 1. Introduction

The weaning of piglets in modern pig production is generally done at an early age and is associated with stresses due to major changes in diet, environment and social groups. Consequently, weaned piglets experience a reduced feed intake, intestinal and immune dysfunction, as well as increased risk of infection with enteric pathogens [1,2]. Thus, the production efficiency of the piggeries is eventually reduced due to the stunted growth, post-weaning diarrhoea and increased mortality of the piglets [3]. 

In the past, post-weaning diarrhoea and weaning related intestinal disorders in pigs were kept in check using in-feed antibiotics, which were banned by the European Union (EU) in 2006 [4] due to the increased risk of antibiotic resistance associated with use of antibiotics in animal feed. Since then, pig producers in Europe have, to a large extent, relied heavily on pharmacological doses of zinc oxide (ZnO) to control post-weaning diarrhoea, but, due to environmental concerns [5] and the risk of antibiotic resistance associated with its uses [6], the EU passed a new law, which suspends the use of pharmacological doses of ZnO from 2022 [7]. Thus, there is an urgent need to find suitable alternative strategies to high doses of in-feed ZnO to sustain the performance and gut health of weaner piglets in countries such as Denmark, where ZnO is still widely used.

Currently, the dietary addition of feeds pre-fermented with probiotic bacteria has attracted attention for its ability to improve production performance and gut health in both pig and poultry production. Fermented feeds have been reported to improve animal performance and gut morphology [8,9], and enhance the immunity of animals [10,11]. They have been also shown to modulate the gut microbiota (GM), partly by the selective inhibition of intestinal pathogens [12,13,14], improve nutrient digestibility and neutralization of anti-nutritional factors in the feed [13,15,16]. Studies involving the feeding of *Lactobacillus* spp. pre-fermented rapeseed meal (FRM) to broiler chickens [8,17] and turkey [18] enhanced growth performance, reduced *Salmonella typhimurium* in the gut, increased lactobacilli counts in excreta, and improved intestinal histomorphology [18]. Similarly, feeding FRM to pregnant sows increased piglet production performance, increased immunoglobulin contents in colostrum, modulated the faecal microbiota in favour of beneficial microbes, and increased nutrient digestibility [19].

Macroalgae-derived products are alternative feed additives with antimicrobial properties, which have attracted attention as potential promoters of gut health. Brown macroalgae, such as *Ascophylum nodossum* (AN) and *Sacharina latissima* (SL), contain wide range of compounds not found in terrestrial plants, e.g., laminarin, fucoidan, and alginate, which have different biological functions, including the ability to modulate gut health, due to their prebiotic and antimicrobial effects [20,21]. 

However, scientific documentation on the impact of these alternative feed additives on weaner piglet performance and health is only available from a rather limited number of feeding trials, and results are not consistent. This variation could be, to a large extent, attributed to large differences in the age of studied piglets and duration of supplementation, as well as large differences in the procedures and bacteria used in the pre-fermentation process. Furthermore, no studies have, to our knowledge, been conducted to investigate whether a potential synergistic effect on performance and gut health could be obtained by including macroalgae in the lactic acid bacteria pre-fermentation of a feed. 

We hypothesized that: (1) the dietary addition of FRM improves weaner piglet performance and gut health due to stimulation of intestinal development and favourable changes in gut barrier function and GM composition; (2) inclusion of the macroalgae AN alone or in combination with SL in the pre-fermentation process has a synergistic effect with FRM on piglet performance and health. The objective of this study was, therefore, to conduct a feeding trial, where piglets from weaning (28 days of age) until approximately 85 days of age were allocated to different dietary additives in their basal diet (inclusion relative to dietary dry matter; DM): (1) NC: negative control, i.e., basal feed with no gut-health-promoting additives, (2) PC: positive control, i.e., basal feed supplemented with 2500 ppm in-feed ZnO, (3) FRM: basal feed with 10% in-feed FRM, (4) FRMA: basal diet with 5% pre-fermented mix of FRM and AN, (5) FRMAS: basal diet with 10.5% pre-fermented mix of FRM, AN and SL. Piglet performance (growth rate, feed efficiency and number of pigs concluding the trial period without antibiotic treatment) was evaluated throughout the experimental period. Feed impact on intestinal development, GM composition and blood biochemistry were assessed in subgroups of 10 piglets per treatment group, sacrificed three weeks after weaning.

## 2. Materials and Methods

### 2.1. Preparation and Formulation of Lactic Acid Bacteria Pre-Fermented Feeds

Lactic acid bacteria (LAB) pre-fermented feeds were obtained from European protein (Baekke, Denmark). Feeds were fermented via solid state fermentation using an inoculum consisting of three lactic-acid fermentative bacteria, namely, *Pediococcus acidilactici* (DSM 16243), *Pediococcus pentosaceus* (DSM 12834) and *Lactobacillus plantarum* (DSM 12837). The addition of the inoculant controls the fermentation process by acidifying the blend within the first 24 h, thus guaranteeing an almost complete anaerobic process. The FRM (commercial name EP100i) was made in a one-step fermentation process, where ground rapeseed meal (80% on DM basis) was mixed with the inoculant broth with constant stirring, followed by decanting and incubation at 38 ℃ for four days. For FRMA (commercial name EP1109) and FRMAS (commercial name EP1199), the fermentations were carried out in a two-step process, where the ground macroalgae was first fermented with the inoculant, and then rapeseed meal was blended into the fermentation, and fermentation was continued for a total of 11 days at 38 ℃ (patent no. WO2008006382A1). The fermented materials were then dried in a spin flash dryer at a temperature setting and pass-through speed that preserve both the viable bacteria as well as the temperature-labile microbial bioactive metabolites (patent no. WO2013029632A1).

### 2.2. Study Animals and Experimental Design 

The feeding experiment was conducted at a commercial pig farm (Kawiks Farm, Patoki 23. 98–170 Widawa. Province. Lodz city, Poland) from May to December 2018. The study involved a total of 1240 piglets entered into the weaner unit (28 days of age) and fed the experimental diets until 85 days of age (eight weeks after weaning). The piglets were born on the farm and recruited from a total of 108 Landrace-Yorkshire crossbred sows (Danbred, Denmark) in their 2nd to 4th parity which farrowed in five different weeks. All litters were standardized to 14 piglets per sow. In each of five weaning weeks, groups of 28 day old piglets were earmarked and transferred to one slatted floor pen in the weaner unit, so that each pen would hold a total of 45–55 piglets from four sows (11–14 piglets per sow, excluding runts). Pens were then randomly assigned to one of five dietary treatments; NC: negative control, consisting of a basal diet without any gut-health-promoting feeds, PC: positive control, consisting of the basal diet supplemented with 2500 ppm ZnO, FRM: basal diet supplemented with 10% FRM (replacing soybean meal) diet on DM basis, FRMA: basal diet supplemented with 5% FRMA (replacing soybean meal) on DM basis, FRMAS: basal diet supplemented with 10.5% FRMAS (replacing soybean meal) on DM basis. Zinc oxide was supplemented only in pre-starter diet and fed until 49 days of age (three weeks post-weaning), while all other dietary supplements were provided with both pre-starter and starter diets throughout the experimental period (until 85 days of age). Each pen represented one experimental unit (replicate). In total, there were four, six, six, five and six replicates of the NC, PC, FRM, FRMA and FRMAS treatments, respectively, distributed over the five insertion weeks.

Three weeks after weaning, i.e., at 49 days of age, subgroups of 10 piglets per treatment (equally distributed on two insertion weeks, and hence slaughter weeks) were sacrificed for acquisition of gastrointestinal organs, tissues and digesta, as well as blood samples. For statistical analyses carried out on those piglets, the piglet was considered as the experimental unit. For records of growth, feed intake and completion rates (see below), the pen was considered the experimental unit. 

### 2.3. Feeding and Recording of Piglets Performance

The piglets received pre-starter diets from weaning (28 days of age) to 49 days of age and starter diets from 50 to 85 days of age, formulated with the specified feeding regimes. The diets were produced and chemically analyzed at the commercial and certified feed factory Wola Pasze Sp.zo.o. (Biała Podlaska, Poland). All diets were formulated to be isoproteinic and isoenergetic across treatment groups (Table S1). Feed and water were available ad libitum from feeding troughs and drinking nipples, respectively, throughout the experimental period. No antibiotics were added to diets or water at any timepoint during the experiment. When piglets manifested signs of diarrhea or other symptoms of serious ailment, they were recorded, removed from the pen and treated elsewhere, and this was continued from weaning to until 77 days of age. Then, the percent of piglets that completed the experiment was calculated by dividing the number of piglets that attended the experiment until 77 days of age (or other durations of interest) by the total number of piglets allocated to each treatment at weaning and multiplying the quotient by 100%. The 10 piglets (five piglets per treatment per each of two slaughter weeks) sacrificed three weeks after weaning were excluded in the assessment of piglets completing the experiment.

Feed intake and piglets’ body weight (BW) were measured at pen level on daily and weekly bases, respectively. The BW at the day of insertion into the weaner unit (start of the experiment) and exit from the experiment (at 85 days at age) were also recorded at pen level. 

### 2.4. Blood and Intestinal Samplings 

In two of the five insertion weeks, subgroups of five piglets (in total, 2 × 5 = 10 piglets per treatment group) were randomly sampled from each of the five treatment groups (pens) on day 21 post-weaning (49 days of age) and transported to a slaughter facility. The body weight of the piglets was measured, and piglets were then sacrificed by electrical stunning followed by de-bleeding. Blood samples were collected immediately during de-bleeding from the jugular vein, using EDTA tubes for the separation of plasma for subsequent haematological analyses and plain tubes for whole blood biochemical analyses. 

After opening the abdominal cavity, the cardiac, pyloroduodenal, ileocecal and anorectal junctions separating the gut sections were ligated, and the stomach, small intestine and large intestines were then removed from the abdominal cavity. Each of the gut segments, with and without contents, were weighed. Tissue samples of 2 cm length were excised from the middle of the small intestine and from the apex of the spiral of the ascending colon. The tissue samples were gently flushed with saline to remove intestinal contents and then placed in 10% buffered formalin and stored at ambient temperature. About 2 cm^3^ of colonic digesta samples were collected in sterile test-tubes containing RNA later^TM^ (Sigma-Aldrich), and stored at room temperature for no longer than 24 h, and then stored at −80 ℃ until analyses.

### 2.5. Intestinal Morphometry and Histopathology

Tissue samples from mid-jejunum and colon were fixed in 10% buffered formalin and delivered to a commercial laboratory (ALAB Weterynaria, Warsaw, Poland) for further processing and an assessment of histomorphometry and histopathological changes. Thus, fixed tissue samples were dehydrated in graded ethanol and xylene baths, embedded in paraffin wax and sliced into 3–4 µm thickness tissue sections, which were then mounted on microscopic slides, stained with haematoxylin and eosin, and assessed for histomorphometrical features and histopathological changes. A standard light microscope Olympus BX41 and CellSens software (Olympus Corporation, Tokyo, Japan) was used for analyses of all histomorphometrical features (jejunal villus height, jejunal crypt depth, jejunal enterocyte height and colon mucosal thickness) and histopathological changes (presence of intra-epithelial lymphocytes, stromal lymphocytes and gut-associated lymphoid tissue (GALT) structures), and all the analyses were done in a blinded fashion. Ten replicate measurements for each of the 10 piglets per dietary treatment were taken and averaged for statistical analysis. Villus height was measured from the tip of the villus to the villus–crypt junction. Crypt depth was measured from the base upwards to the villus–crypt junction. Villus height-to-crypt depth ratio was calculated from crypt depth and villus height. Histomorphological examination and photographic documentation were made for jejunal villus height and crypt depth (at 10× magnification objective lens and eyepiece), jejunal epithelial height, intra-epithelial lymphocytes and stromal lymphocytes infiltration (40× objective lens and 10× eyepiece) and brush border integrity (100× objective lens and 10× eyepiece). The density of GALT structures was evaluated by counting the number of lymphoid follicles visible in 10 fields of view at 4× magnification [22]. The intra-epithelial lymphocyte infiltration was evaluated based on the following scoring scale: normal, slight, moderate, and severe, corresponding to 0–1, 10–15, 15–20 and >20 lymphocytes/100 enterocytes, respectively. For stromal lymphocytes, the scoring was based on visual assessment, as follows: normal (no deviation from the norm), low (slight infiltration, but no damage to the stroma or epithelium), moderate (signs of weak inflammation with some disruption to epithelial continuity and intestinal blood-barrier), severe (moderate inflammation with damage to the epithelium and intestinal blood-barrier).

### 2.6. Blood Hematology, Blood Biochemistry and Serum Immunoglobulin Analyses

Whole blood samples were used for analyses for blood haematological parameters and for the separation of serum for immunoglobulin determinations. EDTA-stabilized plasma samples were used for biochemical analyses. All analyses were done at the ALAB Weterynaria (Warsaw, Poland). Hematological analysis included red blood cell counts (RBC), hematocrite (Ht) value, hemoglobin (Hb) content, red cell distribution width measured by coefficient of variation (RDW-CV), white blood cells counts, differentiation leucocytes count (lymphocytes, monocytes, basophiles, eosinophils and neutrophils) and total platelet counts, and performed using a Sysmex XT 2000i analyzer (Sysmex Corporation, Kobe, Japan). Commercially available, swine-specific ELISA kits were used for the quantification of serum immunoglobulin G (IgG; Cusabio®, Houston, TX, USA) concentrations, according to protocols provided by the manufacturer. Glucose, total cholesterol (TCH), high-density lipoprotein (HDL), low-density lipoprotein (LDL), total triglycerides (TG), phosphorus, total protein (TP), blood urea nitrogen (BUN), uric acid, lactate dehydrogenase (LDH), aspartate aminotransaminase (ASAT), alanine amino-transferase (ALAT) and lysozyme were analysed by respective kits on a Cobas 6000® c501 module (Roche Diagnostics, Indianapolis, IN, USA).

### 2.7. DNA Extraction and Sequencing of 16S rRNA Genes for the Colon Microbiome 

Total DNA was extracted from the colon digesta using Bead-Beat Micro AX Gravity Kit (A&A Biotechnology, Gdynia, Poland) according to the instructions of the manufacturer. The prokaryotic microbial community was characterized using 16S rRNA gene amplicon profiling of the V3-region (Illumina NextSeq-based), as described previously [23].

### 2.8. Data Analysis

Statistical analysis of pen level piglet performance, and results for individual piglets sacrificed three weeks after weaning (gut histomorphology and blood parameters) were analyzed using Linear Mixed Models in R version 3.6.0 [24]. The effects of dietary treatments on the pen level performance of piglets were evaluated using the following statistical model

(1) $$Yijkl=µ+T_{i}+\left(W+W^{2}{)}_{j}+R_{k}(R_{l}\right)+\varepsilon_{ijkl}$$

where Yijkl is the response variable, µ is the overall mean, $T_{i}$ is the effect of dietary treatments (*i* = 1, 2, 3, 4, 5), ${\left(W+W^{2}\right)}_{j}$ is the linear or quadratic effect of the average piglet body weight at weaning (28 days of age) (*j* = 1, 2, 3,…,6), $R_{k}(R_{l})$ is the effect of repetition (*k* = 1, 2, 3, 4, 5, 6) nested within insertion week (*l* = 1, 2, 3, 4, 5), and $\varepsilon_{ijkl}$ is the residual. Treatment was considered as fixed effect, while repetition (nested within insertion week) and insertion weeks were considered as random effects.

Data generated from individual piglets sacrificed three weeks after weaning were analyzed using the following statistical model

(2) $$Yijkl=µ+T_{i}+S_{j}+\;{\left(TxS\right)}_{ij}+B_{k}\;+P_{l}+\varepsilon_{ijkl}$$

where Yijkl is the response variable, µ is the overall mean, $T_{i}$ is the effect of dietary treatment (*i* = 1, 2, 3, 4, 5), $S_{j}$ is the effect of the sex of the piglet (*j* = male or female), ${\left(TxS\right)}_{ij}$ is the treatment and sex interaction, $B_{k}$ is the week of sacrifice as a covariate (*k* = 1, 2), $P_{l}$ is the random effects of individual piglets (*l* = 1, 2, 3, …10) and $\varepsilon_{ijkl}$ is the residual. Treatment and sex were fixed effects, whilst week of sacrifice and individual piglet number were included as a covariate and random effect, respectively. 

Normal distribution of data was tested using normal quantile–quantile (Q–Q) plots. All non-normal data (mostly those of blood biochemistry, i.e., LDH, Lysozyme, TGs, Urea, Uric acid, ALAT and ASAT) were log transformed before statistical analysis. The best fitting model was recruited based on Akaike information criterion (AIC) value. All results are expressed as least-square means (LSM) and standard error of the mean (SEM), and significance was considered at *p* < 0.05.

For microbiome analysis, the raw sequencing reads were merged and trimmed. Chimeras were removed and zero-radius Operational Taxonomic Units (zOTUs) constructed using the UNOISE algorithm implemented in V-search [25,26]. The Green genes (version 13.8) database was used as a reference for annotation. QIIME 2 [27] was used for further analysis. Rare zOTUs, with a frequency below 0.1% of the minimal sample depth, were removed, and the filtered zOTU table was rarified to an adequate sample depth (24,000 reads) for alpha and beta diversity calculations. Principal coordinate analysis (PCoA) was conducted on unweighted and weighted Unifrac distance metrics, and permutational multivariate analysis of variance (PERMANOVA) was performed to detect statistical differences between the groups (all *p* values were Benjamin–Hochberg corrected after pairwise comparison). Specific taxa comparison among groups was analyzed by analysis of the composition of microbiomes (ANCOM) and default settings in QIIME 2 were used to test for statistically significant differences. For pairwise comparison of the different taxa, the Wilcoxon test from rstatix (R package) was adopted to compute *p* values, which were adjusted by the Benjamin–Horchberg method. 

## 3. Results

### 3.1. Piglet Performance

There were no significant differences between piglets in different treatment groups at weaning. As shown in Table 1, PC piglets achieved a numerically, but not significantly, higher BW by the end of the experimental period at 85 days of age compared to NC piglets, and hence no differences were observed between PC and NC piglets, neither for ADG nor FCR. Piglets supplemented with FRM and FRMA after weaning had similar ADG during the experimental period (28–85 days of age) and achieved equally high body weights at 85 days of age as the pigs from the PC group. The FRM-supplemented piglets performed significantly better than NC piglets, despite having the lowest ADG during the first 14 days after weaning. In piglets supplemented with FRMAS, ADG and body weights at 85 days age were not improved over the NC, and they had poorer performance compared to PC and FRM piglets (Table 1).

There were no differences in FCR or piglet completion rates between any of the feeding regimes. The completion rate during the weaner period was generally high in all groups, but numerically lowest in FRMAS (87%) and NC (89%) and numerically highest in FRM (95%) and PC (94%). Positive associations were found between average piglet BW at weaning compared to average BW of piglets at all ages (linear effect), ADG (quadratic effect) and FCR (linear and quadratic effects) during the first three weeks after weaning (Table 1), but not with completion rate at any time during the weaner period.

### 3.2. Gut Development and Morphology

Dietary treatments and sex had no effect (*p* > 0.05) on tissue weights of any of the gut segments or accessory digestive organs, except for a treatment*sex interaction for pancreas weight (*p* = 0.017), where FRMAS and NC males had lower weights compared to NC females and with all other groups in between (Table S2). Differences were observed in STTW (*p* = 0.035), HGTW (*p* = 0.003) and PNW (*p* = 0.008) between piglets sacrificed in the first (five piglets) compared to second (five piglets) batch (Table S2).

**Figure 1:**
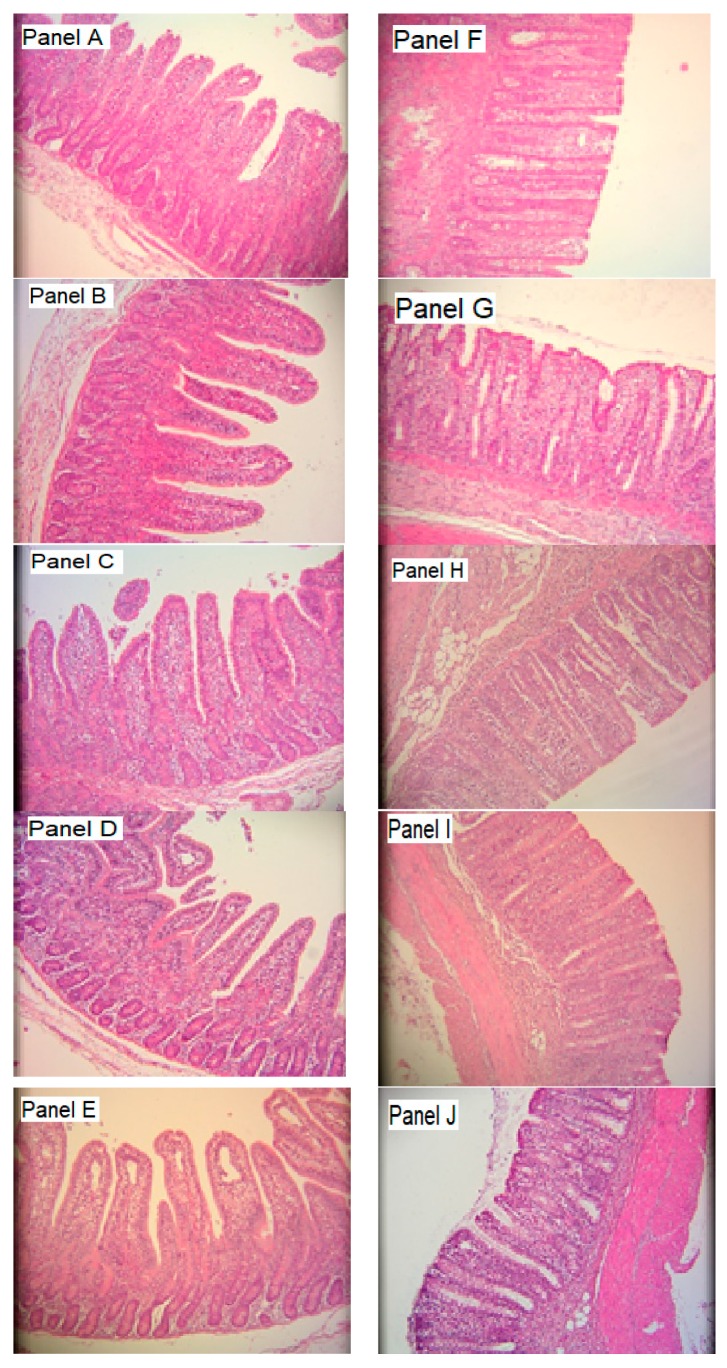
Effects of dietary additives on the histo-morphological changes in the jejunal (left-hand side panels) and colon (right-hand side panels) tissues in piglets 21 days after weaning. (Panels **A**–**E**) in the left hand side column illustrate the corresponding histomorphometry of jejunum tissue from NC, PC, FRM, FRMA and FRMAS groups whereas the left hand side panels, namely (panels **F**–**J**), are an histomorphometry of colon tissue from the NC, PC, FRM, FRMA and FRMAS, respectively. NC, PC, FRM, FRMA and FRMAS are defined as footnote (See legends for Table 1). Histo-micrographs were taken at 10× magnification.

The morphological characteristics of intestinal tissues obtained from piglets in different treatment groups are illustrated in Figure 1, and the impacts of dietary supplementation on morphological features are shown in Table 2. Jejunal villi height (JVH) was increased with supplementation with ZnO (PC) and FRMAS in all piglets, whereas FRM and FRMA increased JVH in male, but not female, piglets. Jejunal crypt depth (JCD) was increased by supplementation with all fermented feeds, except FRMAS in males compared to NC and PC males, but was not affected by diets in females. Villus-to-crypt ratio (VCR) was increased in PC and FRMAS groups, with the highest levels being observed in FRMAS males. Compared to the respective NC males or females, colon mucosal thickness (CMT) was increased and decreased in PC females and males, respectively, and increased by FRM in both sexes, but decreased in FRMAS males. 

**Figure 2:**
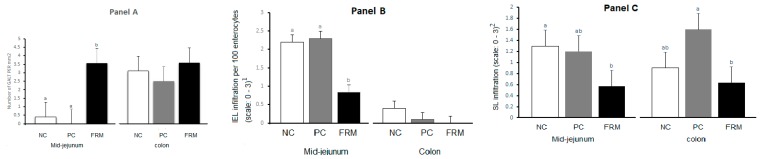
Effects of dietary supplements on the development of gut-associated lymphoid tissue (GALT) structures (Panel **A**), and intraepithelial lymphocytes infiltration (IEL; Panel **B**) and stromal lymphocyte infiltration (SL; Panel **C**) of mid-jejunum, and colon in piglets sacrificed 21 days after weaning. NC, PC, FRM. See legends for Table 1. ^1^ Explanation for IEL score 0–3: 0—normal (0–10 IELs/100 enterocytes); 1—low (10–15 IELs/100 enterocytes); 2—moderate (15–20 IELs/100 enterocytes) suggesting subclinical inflammation; 3—severe (> 20 IELs/100 enterocytes) indicating chronic inflammation.^2^ Explanation for SL score 0–3: 0—normal, presence of single lymphocytes in stroma of villus and crypts; 1—low, presence of an increased number of lymphocytes that do not obscure and damage the stromal structures; 2—moderate, presence of abundant lymphocyte infiltration of the stroma characterized by damaged blood vessels and connective tissue fibres, and reduced visibility stromal structures; 3—severe, excessive lymphocyte infiltration which completely disrupts and conceals the stroma. ^a,b^ Different letters indicate significant difference at *p* < 0.05.

Histopathological evaluation of tissues was performed only for the NC, PC and FRM groups. FRM piglets had more than 8-fold higher numbers of GALT structures in mid-jejunal tissues compared to NC and PC piglets (*p* = 0.012) but demonstrated no significant difference in the number of GALT structures or levels of IEL in the colon tissues (*p* > 0.05; Figure 2A,B). With respect to signs of focal inflammation, FRM piglets had > 60% reduction in scores for intraepithelial lymphocytes (IEL) infiltration in mid-jejunal tissue (*p* < 0.0001; Figure 2B), and > 40% and >30% reduction in stromal lymphocyte (SL) infiltration in mid-jenunal and colon tissues, respectively (*p* < 0.05; Figure 2C). There were no significant differences between NC and PC piglets for these traits. In NC and PC piglets, compared to FRM piglets, the surface of the mid-jejunal epithelium was more uneven, and the brush border was lower and more discontinuous in both mid-jejunum and the colon. Piglets from NC and PC also had signs of mid-jejunal degenerative changes in epithelial continuity and enterocytes in areas characterized by lymphocyte infiltration, indicating a slight-to-moderate state of subclinical inflammation, which was not observed in FRM fed piglets.

### 3.3. Blood Haematology, Blood Biochemistry and Serum Immunoglobulin

Red blood cell (*p* = 0.019), leucocyte (WBC; *p* = 0.001) and neutrophil (p = 0.03) counts were reduced in piglets supplemented with FRMA (except for neutrophils) and FRMAS as compared with NC piglets (*p* < 0.05), and with other groups in between, whereas eosinophil % (*p* = 0.003) and counts (*p* = 0.042) were increased in FRMAS compared to NC and FRM supplemented piglets (Table S3). Female piglets in general had higher eosinophil %, RBC count, HCT % and Hb concentration (*p* < 0.05) compared to male piglets. All blood biochemistry parameters were unaffected by dietary supplementation and sex (Table S3).

### 3.4. Colon Microbiome

The number of observed zOTUs did not vary significantly (*p* > 0.05) across dietary treatments. However, Pielou’s evenness index was significantly increased in all piglets fed fermented feed supplements compared to the NC, but was similar to the PC piglets. Similarly, the Shannon diversity index was significantly higher for FRMA and FRMAS compared to NC, but only FRMA was significantly higher than PC (Table 3). 

**Figure 3:**
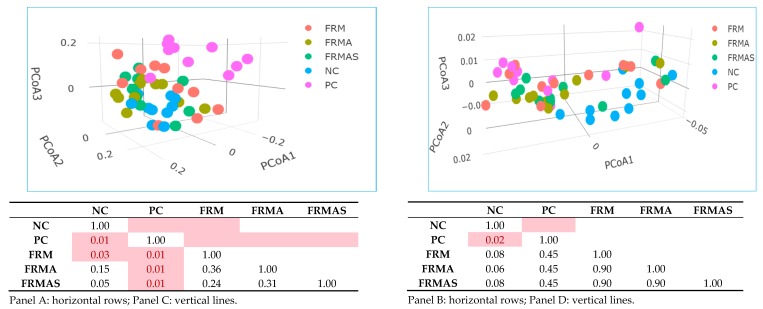
Effects of dietary supplements as determined by unweighted Unifrac (Panel **A**; R^2^ = 0.13, *p* = 0.001) and weighted Unifrac (Panel **B**: R^2^ = 0.18, *p* = 0.022) distance metrics based on the 16S rRNA gene V3-region amplicon sequencing of colon microbiota from piglets sacrificed 21 days after weaning. The *p* values (Benjamin-Hochberg corrected) from pairwise PERMANOVA tests between experimental groups (Panel C, Unweighted and Panel D, Weighted Unifrac distance metrics). Adjusted *p* values below 0.05 highlighted in red. NC (blue dots), PC (purple dots), FRM (orange dots), FRMA (dark green dots), FRMAS (light green dots): NC = negative control with no dietary supplements; PC = positive control with 2500 ppm in-feed ZnO during the first 21 d of the test; FRM = pre-fermented rapeseed meal at 10% of dietary DM inclusion; FRMA = co-pre-fermented rapeseed meal and *Ascophyllum nodossum* at 5% of dietary DM inclusion; FRMAS = co-pre-fermented rapeseed meal, *A. nodossum* and *Saccharina latissima* at 10.5% dietary inclusion.

Generally, Unweighted Unifrac distance metrics-based analysis showed bigger differences between the experimental groups than Weighted Unifrac distance analysis (Figure 3). This indicates that the GM differences between the groups are mainly driven by relatively low abundant taxa, whereas the major taxa only differ between the groups to a lesser degree. Interestingly, Unweighted Unifrac distance analysis showed that PC was significantly different from all other groups, indicating the ZnO induces numerous smaller changes in the GM compared to NC, FRM, FRMA and FRMAS piglets. NC and FRM also separated significantly, while FRMAS was borderline different from NC. However, when focusing on the more abundant taxa (Weighted Unifrac-based), NC differed significantly from PC, and was borderline different from FRM, FRMA and FRMAS (Figure 3).

**Figure 4:**
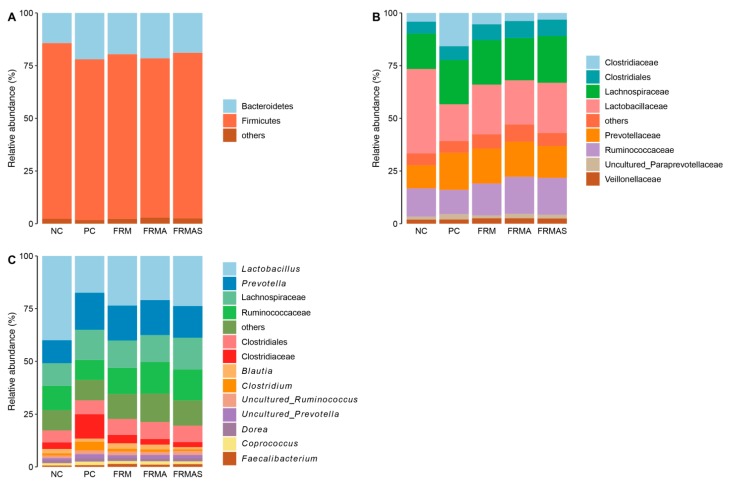
Taxonomic classification of the colon microbiota into the phylum (Panel **A**), family (Panel **B**) and genera (Panel **C**), based on 16S rRNA gene V3-recion sequencing. NC, PC, FRM, FRMA and FRMAS: see legends to Table 1.

The colon microbiota was dominated by the phylum Firmicutes followed by the phylum Bacteroidetes with a negligible contribution from other phyla, irrespective of the dietary treatments. The Firmicutes member, *Lactobacillus* spp., had a high relative abundance (NC = 33.0%, FRMAS = 24.0%, FRM = 23.5%, FRMA = 18.2%, PC = 18.1%) and was the most predominant genus in all treatment groups (Figure 4). *Prevotella* spp., known to play a role in the digestion of complex plant polysaccharides, were also abundant in all groups (PC = 16.9%, FRM =16.6%, FRMA = 16.4, FRMAS = 16.1%, NC = 8.7%). Only two species were found to be significantly different between the experimental groups, as determined by ANCOM analysis, namely *Paludibacter* spp. and *Porphyromonas* spp.: both with a maximum relative abundance of less than 0.5%. These two species were most abundant in NC piglets, and least abundant among PC piglets, with the FRM, FRMA and FRMAS in between (Figure S1). 

## 4. Discussion

Fermented feeds based on protein sources (primarily soybean products) have, in previous studies, been reported to improve performance and gut health in weaned piglets [28,29,30] and poultry [8,17,31]. Certain brown macroalgae species have also been reported to have gut-health-promoting effects [32], but a low digestibility [33,34], which may limit their inclusion in diets for very young animals. To the best of our knowledge, no studies have previously investigated the effects of a dietary additive for weaner piglets, where a protein source is pre-fermented in combination with macroalgae using a mix of lactic acid bacteria (LAB). We speculated that exposure of macroalgae to a pre-fermentation process could increase both the digestibility of the macroalgae and promote the formation or liberation of gut-health-sustaining bioactive components [35]. In this study, we therefore investigated whether weaner piglet performance, gut development and a healthy intestinal microbiota profile can be sustained when in-feed medicinal zinc oxide is replaced by rapeseed meal pre-fermented with LAB alone (FRM diet) or in combination with one (FRMA diet) or two (FRMAS diet) different brown macroalgae species, namely *A. nodossum* and *S. latissima*. 

Rapeseed meal was chosen as the protein feed for pre-fermentation due to an increased focus in Denmark on locally produced protein feeds, and due to previous reports of improved performance (ADG and FCR), when fermented rapeseed meal was fed to growing pigs [16], broiler chickens [8,17,30], ducks [36] and turkeys [18,37]. Consistent with these previous studies, we found an overall best performance indicators during the weaner period in piglets fed a diet with an FRM supplement. In this group, piglets were around 2.5 kg heavier than NC and almost 1 kg heavier than PC piglets at 85 days of age. FRM piglets had a higher ADFI and ADG across the whole experimental period and numerically equal completion rates to PC piglets. Performance indicators of FRMA-supplemented piglets were almost equivalent, and not significantly different, to the performance of FRM piglets. However, the FRMAS supplementation did not improve their performance over that of non-supplemented NC piglets. These differences in performance could not be ascribed to the overall nutritional qualities of the diets, which, across pre-starter and starter diets, were isoproteic, with identical contents of essential nutrients. All diets containing FRM, with or without macroalgae, had higher crude fiber and lower starch, but also higher fat contents, than NC and PC diets, resulting in all diets being isoenergetic (ME). Rapeseed meal and dietary crude fiber is generally believed to have a negative impact on both feed intake and daily weight gains for weaner piglets, but this was not observed in this trial, where rapeseed meal had been subjected to pre-fermentation. It is likely that the controlled microbial pre-fermentation process may have improved the nutritional value of rapeseed meal, since microbial fermentation in other studies has been shown to improve digestibility and neutralize anti-nutritional factors, such as tannins, glucosinolates and phytic acid, in feeds like rapeseed meal [16,38]. 

The general picture regarding dietary impact on gut development was that ZnO (most clearly observed in females) and FRM (most clearly observed in males) stimulated jejunal villi development while ZnO also enhanced the villi-to-crypt-depth ratio in both sexes. These are desirable changes associated with the increased absorptive capacity of the gut [39], which could be implicated in the superior performance of FRM-fed piglets in this trial. The pre-fermentation of rapeseed provides piglets not only with the probiotic bacteria added during the pre-fermentation process [40], but also with health-promoting bioactive metabolites generated during the pre-fermentation process [18,19,38,41]. These bioactive compounds include lactate and butyrate [42,43,44], a vast array of phenolic compounds [45,46,47] which are essential for the modulation of the inflammatory response of intestinal mucosa, as well as antimicrobial peptides produced during fermentation [41]. Furthermore, the digestive capacity of the newly weaned piglet can also be aided by carbohydrate-degrading enzymes such as α-galactosidase, β-galactosidase, α-glucosidase, β-glucosidase, and β-glucuronidase derived from beneficial bacteria [48,49], or the activation of endogenous plant enzymes by fermentation [50]. 

It is therefore puzzling to observe that the most extensive jejunal villi development and highest villus-to-crypt ratios, which were observed in the FRMAS-supplemented piglets, were not translated into improved growth performance. In contrast, piglets supplemented with FRMA had an improved growth performance compared to both FRMAS and NC piglets, although there were no (females) or very modest (males) effects of this supplementation on gut development indicators compared to NC piglets. However, we cannot be certain whether or not this discrepancy could be due to differences in the dose of fermented rapeseed and macroalgae provided to piglets. FRMA was included with 5%, and FRMAS with 10.5%, of dietary DM. The different inclusion rates were decided based on concerns regarding an anticipated low fermentability by gut microbes and strong antimicrobial effect of, particularly, *A. nodossum* (based on pilot in vitro fermentation tests; results not shown); we are uncertain whether it would still demonstrate those characteristics after undergoing the pre-fermentation process. 

Relatively few studies have evaluated the impact of dietary supplementation with macroalgae or macroalgae products for weaner piglets. In some studies, no effects were observed on villus height, crypt depth or villus-to-crypt depth ratios, when macroalgae such as *L. hyperborean*, *L. digitata* or extracts from *A. nodossum* and *Laminaria* were included in the diets of weaned piglets [51,52,53]. However, feeding purified macroalgae carbohydrates, such as laminarin, fucoidan or alginates, to weaned piglets has been reported to increase duodenal villus height and crypt depth [54,55], although not consistently [56]. The increment in gut histomorphometric indices, when FRMAS was fed, could be due to the antioxidant effects of such macroalgae components [57,58], which might ameliorate the oxidative stresses commonly seen during the immediate post-weaning period [59] or could also be due to a prebiotic effect of macroalgal components [60]. 

However, the supplementation of weaned piglets with FRMA did not have any favorable effects on any of the determined gut histomorphometric indices in female piglets, and only had a positive effect on jejunal villi height in male piglets compared to NC piglets. As reviewed by Makkar et al. [20], feeding macroalgae at higher doses may result in a depression of animal performance, while the administration of fucoidan and laminarin together, instead of feeding separately, may have antagonistic effects on beneficial gut microbes [61] and intestinal morphology in weaned piglets [62]. Although we cannot rule out whether antagonistic interactions can explain the differences in production performance between FRMA and FRMAS piglets in this trial, factors other than small intestinal villi development and associated absorptive capacity might be responsible for the lack of association between the observed intestinal development and performance in these piglets. 

Histopathological changes in the jejunum and colon were studied in greater detail only for the NC, PC and FRM piglets, where dietary supplementation with FRM resulted in a healthier jejunal mucosal appearance, as revealed by well-developed and continuous epithelium and brush borders as well as a colon with appreciably enhanced mucosal thickness. Such features are indicative of a more efficient gut barrier function throughout. In agreement with this, intraepithelial and stromal lymphocyte infiltration in jejunum were almost negligible in FRM compared to NC and PC piglets, where lymphocyte infiltrations were higher for both controls to the scale of sub-clinical inflammation, with moderate damage to the intestinal epithelial barrier. Furthermore, a more developed mucosal immunity was found in FRM piglets, as evidenced by a marked increase in the GALT structures, which are essential for mounting appropriate innate (reservoir of macrophages) and adaptive (harbors T- and B-lymphocytes) immune responses in the gut in response to invading pathogens or foreign antigens [63,64,65,66].

Results from the present study indicated that the inclusion of macroalgae in the pre-fermentation process might also have a greater impact on systemic immune functions, since piglets supplemented with, particularly, FRMAS and, to a lesser extent, FRMA, had reduced counts of eosinophils and leucocytes in the blood three weeks after weaning, compared to NC piglets. However, blood biochemical parameters, including IgG, and the weight of the spleen were unaffected by dietary supplementations (Table S2). 

Overall, the colon gut microbiome of the piglets showed high relative abundance of *Lactobacillus* and *Prevotella* spp. *Prevotella* spp. have been reported to be involved in the fermentation of plant fibers in the gut [67], whilst *Lactobacillus* spp. are commonly involved in the breakdown of starch rich carbohydrates [68], and are recognized early gut colonizers [69,70]. 

An optimal development of the gut microbiome is extremely important to ensure that the transition at weaning from liquid sow milk to an exclusively solid, and mostly plant-based, diet occurs as smoothly as possible [71]. Interestingly, piglets from all groups receiving the pre-fermented feeds in the present experiment had a more diverse colon microbiota (Pielou’s evenness and Shannon indices; Table 3) compared to the NC group. An increased richness and diversity of the gut microbial community are reported to be indicators for the stability of gut microbial communities and, hence, improved gut health [14]. Rapeseed meal has a relatively high content of crude fibers compared to soybean, which makes it less suitable as feed for young piglets, but these fibers could be utilized by colon bacteria as prebiotics [72,73]. Hence, the feeding of rapeseed meal, even in a non-fermented form, has been demonstrated to increase the diversity of colon microbiota compared to a control diet based on soybean meal [73]. Similarly, macroalgae contain indigestible, prebiotic carbohydrates, which, upon fermentation, may enrich beneficial bacteria in the gut [67,74,75], despite some contradicting results [51].

Overall, Unweighted Unifrac distance metrics showed that the PC group clearly differed from all other groups, with FRM and FRMAS being placed somewhere between NC and PC. This indicates that ZnO influenced the presence of numerous low abundant taxa, but also that FRM and FRMAS tend to drive the low abundant members of the GM in the same direction. This tendency was even more pronounced when taking the relative abundance of the GM members into consideration (Weighted Unifrac distance analysis), where NC differed from all other experimental groups, whereas PC, FRM, FRMA and FRMAS did not differ. This again indicates that all pre-fermented supplements (FRM, FRMA and FRMAS) were able to push the piglet GM in a direction resembling that of ZnO. A previous study has similarly showed that the feeding of fermented feed in growing-fattening pigs resulted in a beneficial shift in the faecal microbiota, which could be an indicator of proper gut health [14]. Our data have demonstrated that all fermented feeds (with or without the inclusion of macroalgae in the pre-fermentation process) were able to change microbiota profiles in a desirable direction. 

## 5. Conclusions

The addition of FRM to the diets of weaner piglets ensured similar (or numerically improved) feed intake, ADG and BW at 85 days as in-feed zinc, and is thus a promising alternative to in-feed zinc for weaned piglets. This could be attributed to the improved development of small intestinal villi and hind gut mucosa, improved gut barrier function in jejunum and colon (mucosal and brush border integrity, the presence of GALT structures and negligible intestinal inflammation indices), and changes in colon microbiota in the direction of larger microbial diversity (robustness). In herds with a poorer health status than the herd where the present experiment was carried out (high completion rates in all groups), this would be expected to also have a beneficial impact on piglet mortality. Further research is needed to unravel the underlying reason for the lack of association between production performance, and gut development and a healthy colon microbiome upon inclusion of macroalgae in the pre-fermentation process. 

## Figures and Tables

**Table 1:** Effect of lactic acid bacteria pre-femented dietary feeds (rapeseed meal with or without macroalgae) on performance and completion rates of weaned piglets.

Parameters	NC	PC	FRM	FRMA	FRMAS	SEM	*p*-Value				
							TG	Body Weight at Weaning		3 Weeks Post-Weaning	
								L	Q	L	Q
Weaning weight (age 28 days), kg	6.07 ± 0.33	5.86 ± 0.98	6.16 ± 0.54	6.21 ± 0.79	5.75 ± 1.03		NS				
Body weight, kg, at age (days):											
42	7.11 ^a^	6.99 ^a^	6.34 ^b^	6.79 ^ab^	6.97 ^a^	0.30	0.050	0.000	0.033		
49	8.21	9.35	8.58	8.13	8.02	0.42	0.191	0.002	0.860		
77	20.13	20.73	20.29	20.30	19.19	0.80	0.317	0.012	0.432		
85	22.42 ^ac^	24.04 ^ab^	24.97 ^b^	23.87 ^bc^	22.26 ^c^	0.72	0.015	0.020	0.134		
ADFI, kg/day, in age interval (days):											
28–42	0.161	0.177	0.170	0.186	0.165	0.018	0.577	0.252	0.732		
28–49	0.223	0.248	0.235	0.238	0.208	0.067	0.464	0.512	0.620		
28–85	0.528 ^ab^	0.527 ^ab^	0.573 ^a^	0.526 ^ab^	0.469 ^b^	0.025	0.030	0.529	0.522		
50–85	0.691 ^ab^	0.698 ^ab^	0.753 ^a^	0.697 ^ab^	0.629 ^b^	0.035	0.033			0.689	0.322
ADG, kg/day, in the age interval (days):											
28–42	0.078	0.075	0.032	0.058	0.067	0.025	0.224	0.616	0.116		
28–49	0.106	0.160	0.123	0.102	0.097	0.020	0.204	0.636	0.859		
28–85	0.293 ^ac^	0.322 ^ab^	0.339 ^b^	0.319 ^bc^	0.290 ^c^	0.013	0.030	0.960	0.135		
50–85	0.413	0.426	0.466	0.444	0.393	0.025	0.077			0.217	0.155
FCR in the age interval (days):											
28–42	3.37	2.39	3.79	2.85	3.13	0.81	0.147	0.009	0.042		
28–49	2.31	1.62	2.22	2.56	2.53	0.44	0.539	0.691	0.858		
28–85	1.75	1.65	1.62	1.66	1.64	1.63	0.430	0.622	0.716		
50–85	1.67	1.65	1.58	1.59	1.58	0.047	0.538			0.346	0.374
Completion rate (%) in the age interval (days):											
28–42	94.63	96.11	94.9	96.74	96.13	3.06	0.977	0.739	0.665		
28–49	92.0	95.4	93.7	97.6	96.4	2.9	0.845	0.743	0.190		
28–77 *	89.0	93.6	95.3	89.9	87.0	4.3	0.442	0.967	0.869		
50–77 *	98.3	96.9	97.7	94.4	94.5	3.2	0.714			0.960	0.833

^a–c^ Different superscript in the row show significance difference between treatments at *p* < 0.05. NC = negative control with no dietary supplements; PC = positive control with 2500 ppm in-feed ZnO during the first 21 d of the test; FRM = pre-fermented rapeseed meal at 10% of dietary DM inclusion; FRMA = co-pre-fermented rapeseed meal and *Ascophyllum nodossum* at 5% of dietary DM inclusion; FRMAS = co-pre-fermented rapeseed meal, *A. nodossum* and *Saccharina latissima* at 10.5% dietary inclusion. ADFI = average daily feed intake; ADG = average daily gain; FCR = feed conversion ratio; TG = treatment group; L = linear effect; Q = quadratic effect; NS = not significantly different (based on t-tests between treatmens) * Data for completion rate were available only until 77 days of age, although the piglets continued the experiment until 85 days of age.

**Table 2:** Effect of dietary supplementation (rapeseed meal, pre-fermented alone or in combination with one or more species of macroalgae) on the morphological features of jejunum and colon in piglets 21 days post-weaning.

Parameters	Female					Male					SEM	*p*-Value			
	NC	PC	FRM	FRMA	FRMAS	NC	PC	FRM	FRMA	FRMAS		TG	Sex	TG × Sex	Week
JVH	398 ^a^	430 ^b^	401 ^a^	380 ^ac^	435 ^b^	364 ^c^	404 ^a^	391 ^a^	402 ^a^	448 ^b^	6.9	0.000	0.003	0.000	0.099
JCD	208 ^ab^	218 ^b^	221 ^b^	209 ^ab^	216 ^b^	191 ^cd^	202 ^ac^	218 ^b^	218 ^b^	180 ^d^	4.4	0.000	0.038	0.000	0.000
VCR	1.97 ^a^	2.18 ^b^	1.87 ^a^	1.84 ^a^	2.16 ^b^	1.99 ^a^	2.18 ^b^	1.95 ^a^	1.94 ^a^	2.48 ^c^	0.05	0.000	0.468	0.002	0.000
CMT	454 ^bd^	549 ^e^	478 ^c^	441 ^ad^	460 ^bc^	455 ^bd^	428 ^af^	514 ^g^	447 ^ab^	416 ^f^	5.7	0.000	0.000	0.000	0.000

^a–c^ Different superscript in the row show significance difference between treatments at *p* < 0.05. NC = negative control with no dietary supplements; PC = positive control with 2500 ppm in-feed ZnO during the first 21 d of the test; FRM = pre-fermented rapeseed meal at 10% of dietary DM inclusion; FRMA = co-pre-fermented rapeseed meal and *Ascophyllum nodossum* at 5% of dietary DM inclusion; FRMAS = co-pre-fermented rapeseed meal, *A. nodossum* and *Saccharina latissima* at 10.5% dietary inclusion: TG = treatment group; TG × Sex = treatment group and sex interaction. JVH = jejunal villi height; JCD = jejunal crypt depth; VCR = villi to crypt ratio; CMT = colonic mucosa thickness. Week refers to the two different weeks where subgroups of piglets (five each time) were sacrificed.

**Table 3:** Alpha diversity indices of colon microbiota of piglets exposed to different dietary additives and sacrificed three weeks after weaning. Values with different superscripts are significantly different.

Alpha Diversity Indices	NC	PC	FRM	FRMA	FRMAS	*p*
Observed zOTUs	3530.5 ± 47.5	3335.2 ± 55.4	3679.6 ± 66.4	3948.5 ± 70.7	3921.4 ± 57.8	0.092
Shannon index	8.65 ^a^ ± 0.08	9.29 ^ab^ ± 0.06	9.52 ^abc^ ± 0.09	9.79 ^c^ ± 0.10	9.53 ^bc^ ± 0.10	0.047
Pielou’s evenness	0.73 ^a^ ± 0.006	0.80 ^b^ ± 0.004	0.80 ^b^ ± 0.006	0.82 ^b^ ± 0.007	0.80 ^b^ ± 0.007	0.019

^a–c^ Different superscript in the row show significance difference between treatments at *p* < 0.05. OTU: operational taxonomic units. NC = negative control with no dietary supplements; PC = positive control with 2500 ppm in-feed ZnO during the first 21 d of the test; FRM = pre-fermented rapeseed meal at 10% of dietary DM inclusion; FRMA = co-pre-fermented rapeseed meal and *Ascophyllum nodossum* at 5% of dietary DM inclusion; FRMAS = co-pre-fermented rapeseed meal, *A. nodossum* and *Saccharina latissima* at 10.5% dietary inclusion.

## Supplementary Materials

The following are available online at https://www.mdpi.com/2076-2615/10/1/137/s1, Table S1. Feed ingredients and chemical composition of diets for weaner piglets. NC = negative control with no dietary supplements; PC = positive control with 2500 ppm in-feed ZnO in the pre-starter diet; FRM = pre-fermented rapeseed meal at 10% of dietary DM inclusion; FRMA = co-pre-fermented rapeseed meal and *Ascophyllum nodossum* at 5% of dietary DM inclusion; FRMAS = co-pre-fermented rapeseed meal, *A. nodossum* and *Saccharina latissima* at 10.5% of dietary DM inclusion., Table S2. Effect of pre-femented dietary additives (rapeseed meal with or without macroalgae) on weights of gut segments and accessory digestive organs of weaner piglets, Table S3. Effect of pre-fermented dietary additives (rapeseed meal with or without) macroalgae on blood chemistry of weaner piglets fed different diets after weaning: NC = negative control with no dietary supplements; PC = positive control with 2500 ppm in-feed ZnO in the pre-starter diet; FRM = pre-fermented rapeseed meal at 10% of dietary DM inclusion; FRMA = co-pre-fermented rapeseed meal and *Ascophyllum nodossum* at 5% of dietary DM inclusion; FRMAS = co-pre-fermented rapeseed meal, *A. nodossum* and *Saccharina latissima* at 10.5% of dietary DM inclusion., Figure S1: Relative abundance of colon microbiota species from piglets sacrificed 21 days after weaning that were found to be significantly different between dietary supplementation as determined by ANCOM analysis. NC = negative control with no dietary supplements; PC = positive control with 2500 ppm in-feed ZnO in the pre-starter diet; FRM = pre-fermented rapeseed meal at 10% of dietary DM inclusion; FRMA = co-pre-fermented rapeseed meal and *Ascophyllum nodossum* at 5% of dietary DM inclusion; FRMAS = co-pre-fermented rapeseed meal, *A. nodossum* and *Saccharina latissima* at 10.5% of dietary DM inclusion.
