# Supplementary material for: Impact of Dietary Supplementation of Lactic Acid Bacteria Fermented Rapeseed with or without Macroalgae on Performance and Health of Piglets Following Omission of Medicinal Zinc from Weaner Diets
Source: Animals (Basel). 2020 Jan 15;10(1):137. doi: 10.3390/ani10010137 (PMC7023219; doi:10.3390/ani10010137)
Supplement: Supplementary file 1 [file animals-10-00137-s001.pdf]

Supplementary Tables

Table S1. Feed ingredients and chemical composition of diets for weaner piglets. NC=Negative control with no dietary supplements; PC=positive control with 2500 ppm *in-feed* ZnO in the pre-starter diet; FRM=Pre-fermented rapeseed meal at 10% of dietary DM inclusion; FRMA=Co-pre-fermented rapeseed meal and *Ascophyllum nodossum* at 5% of dietary DM inclusion; FRMAS=Co-pre-fermented rapeseed meal, *A. nodossum* and *Saccharina latissima* at 10.5% of dietary DM inclusion.

Composition	Pre-starter diets (g/kg DM feed)					Starter diets (g/kg DM feed)				
	NC	PC[1]	FRM	FRMA	FRM AS	NC	PC	FRM	FRMA	FRMAS
Wheat (11.2%)	613.3	603.1	554.4	581.6	551.1	539.9	539.9	481.7	507.2	479.1
Barley (10.6%)	100.0	100.0	100.0	100.0	100.0	200.0	200.0	200.0	200.0	200.0
Fermented feeds			100.0	50.0	105.0			100.0	50.0	105.0
Commercial soybean meal for piglets	79.1	81.2	39.7	62.1	37.7	10.6	10.6			
Soybean meal (46.0%)	-	-	-	-	-	170	170	155.2	169.9	152.7
Potato protein (76%)	40.0	40.0	40.0	40.0	40.0					
Fish meal (70%)	40.0	40.0	40.0	40.0	40.0	14.0	14.0		9.00	
Whey protein 12.5%	50.0	50.0	50.0	50.0	50.0					
Soybean oil	37.3	40.4	43.1	39.9	43.5	23.7	23.7	28.0	26.1	28.2
Limestone (Ca-38.5%)	-	-	-	-	-	5.71	5.71	5.28	5.43	5.34
Calcium formate	5.00	5.00	5.00	5.00	5.00					
Monocalcium Phosphate	9.12	10.7	7.38	8.34	7.28	8.83	8.83	7.73	8.29	7.63
Sodium chloride	3.83	3.83	3.43	3.18	3.38	4.98	4.98	4.89	4.44	4.85
Flavor	2.00	2.00	2.00	2.00	2.00	2.00	2.00	2.00	2.00	2.00
Lysine HCl 98%	5.82	5.77	5.93	5.80	5.92	5.60	5.60	5.98	5.67	5.97
Tetracid (liquid)	5.00	5.00		2.50		5.00	5.00		2.50	
DL-Methionine (99%)	0.77	0.77	0.56	0.69	0.56	1.02	1.02	0.97	0.99	0.97
L-Threonine (99%)	1.88	1.87	1.63	1.78	1.63	1.94	1.94	1.74	1.86	1.74
Valine 98%	1.11	1.10	0.94	1.78	0.94	1.00	1.00	0.86	0.97	0.87
Tryptophan 99%	0.65	0.65	0.64	0.66	0.65	0.37	0.37	0.34	0.36	0.34
[2]Microbial phytase	0.15	0.15	0.15	0.15	0.15	0.15	0.15	0.15	0.15	0.15
[3]Microbial Xylanase, Beta-glucanase		0.15	0.15	0.15	0.15	0.15	0.15	0.15	0.15	0.15

Vitamin E 50%	0.03	0.03	0.03	0.03	0.03	0.01	0.01	0.01	0.01	0.01
[4]Premix 0.5%	5.00	5.00	5.00	5.00	5.00	5.00	5.00	5.00	5.00	5.00
Zinc oxide (78%)	-	3.20	-	-	-	-	-	-	-	-
Calculated nutritional value										
Dry mass%	88.5	88.6	88.6	88.6	88.7	87.5	87.5	87.7	87.6	87.7
Metabolizable energy (MJ)	14.3	14.3	14.3	14.3	14.3	13.5	13.5	13.5	13.5	13.5
Crude protein (%)	19.7	19.7	19.7	19.7	19.7	18.4	18.4	18.4	18.4	18.4
Crude fat (%)	5.56	5.86	6.26	5.88	6.31	4.03	4.03	4.5	4.29	4.52
Crude fiber (%)	2.2	2.18	2.81	2.52	2.87	3.11	3.11	3.77	3.46	3.83
Crude ash (%)	5.37	5.81	5.58	5.5	5.57	5.69	5.69	6.05	5.86	6.05
Starch (g)	417.4	411.3	382.4	398.6	380.4	425.6	425.6	391.1	406.2	389.5
Lactose (g)	36.5	36.5	36.5	36.5	36.5	-	-	-	-	-
Calcium (%)	0.83	0.85	0.86	0.84	0.86	0.82	0.82	0.82	0.82	0.82
Total phosphorus (%)	0.65	0.68	0.65	0.65	0.65	0.58	0.58	0.58	0.58	0.58
Dig. phosphorus (%)	0.59	0.63	0.58	0.59	0.58	0.51	0.51	0.50	0.51	0.50
Sodium (%)	0.23	0.23	0.23	0.23	0.23	0.22	0.22	0.22	0.22	0.22
Chlorides (%)	0.57	0.57	0.56	0.54	0.56	0.51	0.51	0.51	0.49	0.50
Potassium (%)	6.65	0.65	0.68	0.66	0.68	0.67	0.70	0.73	0.69	0.73
Lysine (%)	1.46	1.46	1.46	1.46	1.46	1.28	1.28	1.28	1.28	1.28
Methionine (%)	0.45	0.45	0.45	0.45	0.45	0.41	0.41	0.41	0.41	0.41
Met + Cys (%)	0.77	0.77	0.80	0.78	0.80	0.72	0.72	0.75	0.73	0.75
Threonine (%)	0.92	0.92	0.92	0.92	0.92	0.81	0.81	0.81	0.81	0.81
Tryptophan (%)	0.31	0.31	0.31	0.31	0.31	0.26	0.26	0.26	0.26	0.26
Valine (%)	1.03	1.03	1.03	1.03	1.03	0.90	0.90	0.90	0.90	0.90
Isoleucine (%)	0.78	0.79	0.78	0.78	0.78	0.70	0.70	0.69	0.69	0.69

[1]Medicinal zinc oxide (78%) was included in only pre-starter PC diet at 2500 ppm (2.5 g/kg) and the piglets from that group followed throughout the entire experimental period.

[2]Quantum® Blue – enhanced *E. coli* phytase optimized to breakdown phytate in feed (ABvista).

[3]Axtra® XB 201. Mixture of beta-glucanase and beta-xylanase (Danisco Animal Nutrition – Dupont).

[4]Mineral-vitamin premix in mg/kg of diet for both pre-starter and starter diets: vitamin A – 13,000 IU; vitamin D_3- 2000 IU; vitamin E – 165 mg; vitamin B1 – 2.5 mg; vitamin B2 – 7.0 mg; vitamin B6 – 4.0 mg; vitamin B12 – 0.05 mg; vitamin C – 100 mg; vitamin K – 3 mg; biotin – 0.2 mg; niacin – 35 mg; folic acid – 1.5 mg; pantothenic acid – 21.7 mg; iron – 180 mg; zinc – 150 mg; manganese – 55 mg; selenium – 0.40 mg; iodine – 0.60 mg.

Table S2. Effect of pre-femented dietary additives (rapeseed meal with or without macroalgae) on weights of gut segments and accessory digestive organs of weaner piglets

	Female					Male					SEM	P-value			
	NC	PC	FRM	FRMA	FRMAS	NC	PC	FRM	FRMA	FRMAS		TG	Sex	TG x Sex	Week
STTW	113	109.8	108.2	103.7	95.7	103.4	100.3	98.6	94.1	86.1	7.8	0.469	0.197	NS	0.035
SITW	479	468	519	499	472	502	491	543	522	495	31.8	0.514	0.312	NS	0.503
HGTW	287	279	305	271	270	260	252	278	245	243	22.3	0.802	0.259	NS	0.003
LVW	324	347	332	305	317	308	330	316	289	300	18.4	0.442	0.275	NS	0.692
PNW[1]	29.9[a]	28.3[ab]	21.6[ab]	23.1[ab]	21.6[ab]	19.7[b]	22.1[ab]	28.4[ab]	27.1[ab]	18.0[b]	3.32	0.214	0.221	0.017	0.008
SPW	26.9	31.7	34.2	30.1	31.7	24.2	28.9	31.4	27.3	28.9	3.49	0.483	0.373	NS	0.066

[1]Treatment and sex interaction (TG x Sex) for PNW (P=0.017)

TG = Treatment group; NC=Negative control with no dietary supplements; PC=positive control with 2500 ppm *in-feed* ZnO in the pre-starter diet; FRM=Pre-fermented rapeseed meal at 10% of dietary DM inclusion; FRMA=Co-pre-fermented rapeseed meal and *Ascophyllum nodossum* at 5% of dietary DM inclusion; FRMAS=Co-pre-fermented rapeseed meal, *A. nodossum* and *Saccharina latissima* at 10.5% of dietary DM inclusion. STTW=stomach tissue weight; SITW=Small intestine tissue weight; HGTW= hindgut tissue weight; LVW= liver weight, PNW=pancreas weight; SPW= spleen weight. Week refers to two different experimental weeks, where batches of piglets were sacrificed 21 days after weaning (5 piglets each of the two week).

Table S3. Effect of pre-fermented dietary additives (rapeseed meal with or without) macroalgae on blood chemistry of weaner piglets fed different diets after weaning: NC=Negative control with no dietary supplements; PC=positive control with 2500 ppm *in-feed* ZnO in the pre-starter diet; FRM=Pre-fermented rapeseed meal at 10% of dietary DM inclusion; FRMA=Co-pre-fermented rapeseed meal and *Ascophyllum nodossum* at 5% of dietary DM inclusion; FRMAS=Co-pre-fermented rapeseed meal, *A. nodossum* and *Saccharina latissima* at 10.5% of dietary DM inclusion.

Parameters	Treatments						Sex			P-value		
	NC	PC	FRM	FRMA	FRMAS	SEM	Female	Male	SEM	TG	Sex	Insertion week
**Erythrocyte indices**												
RBC (10^{10}/L)	612[a]	574[ab]	596[ab]	548[b]	548[b]	14.5	593[a]	559[b]	9.24	0.019	0.022	0.112
Hb (g/dL)	10.06	9.67	9.67	9.31	9.44	0.250	9.94[a]	9.32[b]	0.159	0.307	0.013	0.202
Ht (%)	33.4	32.6	32.2	30.1	31.2	0.98	33.0[a]	30.8[b]	0.62	0.177	0.015	0.886
MCV, fL	54.6	57.0	54.0	54.8	57.1	1.27	55.9	55.1	0.81	0.219	0.401	0.138
MCH, pg/cell	16.5	16.9	16.2	17.0	17.2	0.318	16.8	16.7	0.202	0.136	0.575	0.709
MCHC, g/dL	3.41	3.39	3.41	3.44	3.41	0.015	3.41	3.41	0.010	0.252	0.632	0.073
RDW-CV	24	24.4	22.9	22.1	24.3	0.84	23.2	23.9	0.536	0.285	0.466	0.022
ESR, mm/h	1.08	1.48	1.93	1.44	2.02	0.426	1.42	1.76	0.276	0.509	0.385	0.922
**Leucocyte indices**												
Platelets (10^{10}/L)	37.4	41.1	32.0	33.7	29.2	3.48	36.3	33.1	2.21	0.147	0.440	0.064
Leucocytes (10^9/L)	36.4[a]	28.9[ab]	29.1[ab]	25.2[b]	21.6[b]	2.31	29.4	27.1	1.47	0.001	0.111	0.000
Neutrophils (%)	55.1	51.3	60.5	50.1	48.9	4.21	54.8	51.6	2.67	0.378	0.686	0.002
Neutrophils (10^9/L)	19.3[a]	14.9[ab]	17.6[ab]	13.1[ab]	10.5[b]	1.94	16.2	13.9	1.23	0.03	0.185	0.675
Lymphocytes (%)	40.7	44.2	36.6	44.5	46.5	3.72	41.1	43.9	2.36	0.447	0.727	0.000
Lymphocytes (10^9/L)	15.7	12.9	10.7	11.0	10.2	1.58	12.1	12.1	1.00	0.089	0.474	0.000
Monocytes (%)	3.37	3.52	2.54	4.27	3.43	0.78	3.18	3.67	0.49	0.678	0.412	0.290
Monocytes (10^9/L)	1.16	8.97	8.31	1.67	6.58	3.67	1.18	9.05	0.23	0.307	0.442	0.484
Eosinophils (%)	0.169[a]	0.297[ab]	0.183[a]	0.494[ab]	0.575[b]	0.095	0.431[a]	0.256[b]	0.061	0.003	0.05	0.938
Eosinophils (10^9/L)	0.074	0.087	0.058	0.012	0.016	0.029	0.124	0.077	0.019	0.042	0.078	0.919

Basophils (%)	0.540	0.570	0.164	0.320	0.276	0.140	0.310	0.438	0.089	0.204	0.437	0.085
**Blood biochemistry**												
ALAT (U/L)[1]	3.95	4.26	3.87	3.94	4.11	0.154	4.02	4.03	0.098	0.376	0.682	0.000
ASAT (U/L)[1]	4.50	4.67	4.52	4.52	4.94	0.193	4.55	4.71	0.125	0.566	0.606	0.006
BUN (mg/dL)[1]	2.94	2.69	2.39	2.35	2.52	0.158	2.48	2.68	0.100	0.072	0.097	0.041
LDH (U/L)[1]	6.88	7.08	6.97	7.02	7.26	0.156	7.01	7.07	0.099	0.549	0.891	0.001
Uric acid (mg/dL)[1]	0.240	0.290	0.335	0.350	0.595	0.123	0.425	0.299	0.078	0.213	1.49	0.007
TP (g/dL)	4.41	4.67	4.75	4.65	4.69	0.243	4.59	4.68	0.154	0.868	0.527	0.055
Glucose (mg/dL)	102.1	97.6	95.2	92.5	98.6	6.72	101.0	93.4	4.26	0.819	0.324	0.054
Phosphorous (mg/dL)	9.65	9.83	9.28	9.08	10.24	0.651	9.65	9.58	0.413	0.719	0.622	0.005
TCH (mg/dL)	73.0	72.1	66.8	73.1	78.7	5.95	73.5	72.0	3.78	0.682	0.505	0.003
TG (mg/dL)[1]	3.69	3.72	3.67	3.70	3.93	0.145	3.82	3.67	0.092	0.543	0.211	0.261
LDL (mg/dL)	39.9	38.0	35.1	38.5	36.4	3.58	35.9	39.3	2.27	0.899	0.289	0.975
HDL (mg/dL)	34.0	31.5	28.2	30.5	30.2	3.11	31.1	30.7	1.98	0.746	0.819	0.494
**Humoral defense molecules**												
IgG (µg/mL)	2626	2505	2293	2411	2146	135	2369	2423	84.9	0.090	0.554	0.000
Lysozyme (pmol/mL)[1]	5.05	4.96	4.86	4.93	4.89	0.109	4.93	4.95	0.069	0.742	0.387	0.000

RBC = red blood cells, Hb = haemoglobin, Ht = hematocrit, MCV = mean corposcular volume, MCH = mean corposcular heamoglobin, MCHC = mean corposcular heamoglobin concentration, RDW-CV = red blood cell distribution width, ESR = erythrocyte sedimentation rate, WBC = white blood cells, ALAT=alanine-aminotransferase; ASAT= aspartate aminotransaminase, BUN = blood urea nitrogen, LDH=lactate dehydrogenase, TP = total protein, TCH = total cholesterol, TG = total triglycerides, LDL=low density lipoprotein, HDL=high density lipoprotein; IgG = Immunoglobulin G.

[1]log transformed.

Supplementary Figure

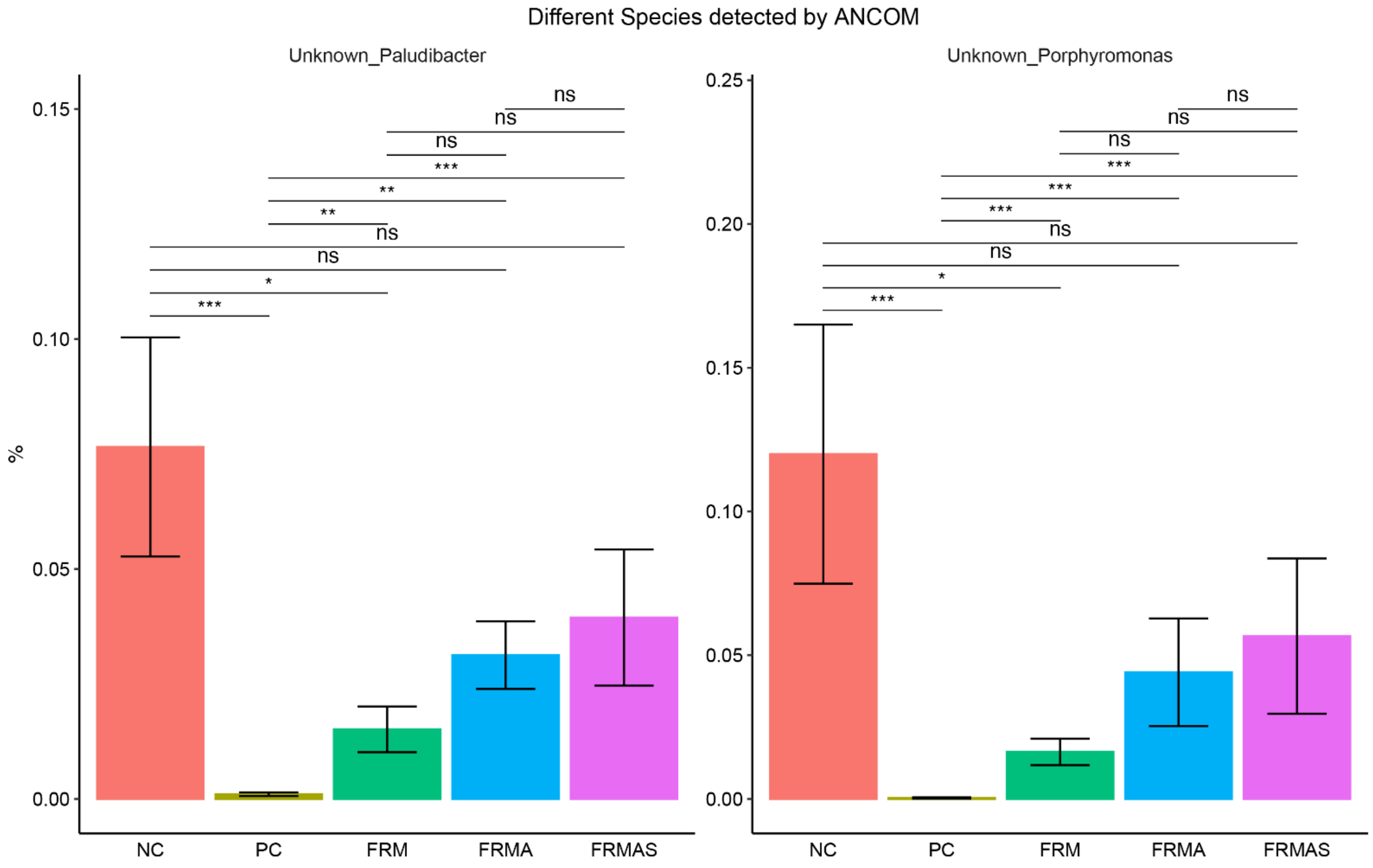

Figure S1: Relative abundance of colon microbiota species from piglets sacrificed 21 days after weaning that were found to be significantly different between dietary supplementation as determined by ANCOM analysis. NC=Negative control with no dietary supplements; PC=positive control with 2500 ppm *in-feed* ZnO in the pre-starter diet; FRM=Pre-fermented rapeseed meal at 10% of dietary DM inclusion; FRMA=Co-pre-fermented rapeseed meal and *Ascophyllum nodossum* at 5% of dietary DM inclusion; FRMAS=Co-pre-fermented rapeseed meal, *A. nodossum* and *Saccharina latissima* at 10.5% of dietary DM inclusion.
